# Mechanism of Response of Watershed Water Quality to Agriculture Land-Use Changes in a Typical Fuel Ethanol Raw Material Planting Area—A Case Study on Guangxi Province, China

**DOI:** 10.3390/ijerph19116499

**Published:** 2022-05-26

**Authors:** Guannan Cui, Xinyu Bai, Pengfei Wang, Haitao Wang, Shiyu Wang, Liming Dong

**Affiliations:** 1 School of Ecology and Environment, Beijing Technology and Business University, Beijing 100048, China; cuiguannan@btbu.edu.cn (G.C.); baixinyu0909@st.btbu.edu.cn (X.B.); wanghaitao008@st.btbu.edu.cn (H.W.); wangshiyu979@st.btbu.edu.cn (S.W.); 2 State Environmental Protection Key Laboratory of Food Chain Pollution Control, Beijing Technology and Business University, Beijing 100048, China; 3 Key Laboratory of Cleaner Production and Integrated Resource Utilization of China National Light Industry, Beijing Technology and Business University, Beijing 100048, China; 4 National Engineering Laboratory for Lake Pollution Control and Ecological Restoration, State Environment Protection Key Laboratory for Lake Pollution Control, Chinese Research Academy of Environmental Sciences, Beijing 100012, China; wangpf01@craes.org.cn

**Keywords:** agricultural crop structures, nonpoint source pollution, energy development, cassava, MIKE-SHE

## Abstract

Speeding up the promotion and application of biofuel ethanol has been a national strategy in China, which in turn has affected changes in the raw material planting structure. This study analyzed the response mechanism of water quality to agriculture land-use changes in a cassava fuel ethanol raw material planting area. The results revealed that an increase in cultivated land and construction land would lead to a rise in the load of TN (total nitrogen) and TP (total phosphorus), while an expansion in forest land and grassland area would reduce the load. As for crop structures, corn would have a remarkable positive impact on TN and TP, while rice and cassava performed in an opposite manner. Furthermore, scenarios under the carbon neutralization policy were carried out to forecast the nonpoint source pollutants based on the quantitative relations coefficients. It was proven that cassava planting was suitable for vigorous fuel ethanol development, but the maximum increase area of cassava should be 126 km^2^ to ensure economic benefits. Under the change in fuel ethanol policy, this study could provide scientific support for local agriculture land-use management in realizing the carbon neutralization vision and also set a good example for the development of the cassava fuel ethanol industry in other cassava-planting countries.

## 1. Introduction

With the development of the global economy from the last century, the human demand for energy has been increasing day by day. In November 2018, the World Energy Outlook issued by the International Energy Agency showed that the global primary energy demand will increase by more than 25% by 2040 [1]. However, the consumption of fossil energy has caused serious harm to the environment, which has experienced more and more serious situations. To ensure sustainable development, China has promised to take on the burden of the responsibility of a big country and achieve the goal of carbon neutralization by 2060. As a clean energy source, biofuel ethanol could replace traditional fossil energy and contribute to sustainable development by reducing greenhouse gas emissions. According to the Paris Agreement in 2021, the widespread use of biofuels could contribute to the goal of reducing greenhouse gases by 80–95% by 2050 [2,3]. In recent years, many countries, such as China, have promulgated a series of supporting policies for the development of biofuels [4]. The global production and consumption of biofuels have increased from 64 billion in 2007 to over 145 billion in 2017 [5]. The development of biofuel ethanol was considered to be one of the ways to achieve carbon neutralization [6]. In order to achieve this goal, China would continue to increase its share of non-fossil energy. Therefore, biofuel ethanol will have a broad development prospect in the future [7].

Due to fuel policy changes, the development of biofuel ethanol would inevitably lead to planting structure variations in raw material crops [8]. Land-use covers had a significant correlation with water quality elements [9]. A large number of researchers have shown that land-use changes have exerted an important impact on nonpoint source pollution in the watershed and that results would guide land-use or cultivation management for local planning [10,11,12,13]. Ni analyzed the response relationship of land-use change to TN and TP in the Great Sunflower River Basin in the US. As the area of the cropland increased, TN and TP yields increased by 12.7% and 10.2%, respectively [14]. Chen found that when land use was changed from cotton to herbaceous perennid, the watershed TN load would decrease by 30–40% [15]. Bioenergy possesses an environmentally friendly advantage, but fuel crops are still land-consuming crops. The application of large amounts of fertilizer during planting will lead to the deterioration of water environments [16]. Liu found that nitrogen loss in planting an ethanol raw crop, corn, was the main cause of nonpoint source pollution in a river basin [17]. The impact of two kinds of biofuel crops in the United States on source pollution has also been studied by Roy. It was found that planting switchgrass instead of corn could greatly improve the water quality of the watershed [18]. Most researchers carried out land-use factors calculations based on the first classifications quoted in the land-use classification GBT21010-2017. There was a lack of consideration of specific crop structures, especially fuel ethanol raw material crop transformations, in past studies. In the future, research on nonpoint source pollution will gradually transition to fine simulation and management at filed scales [19].

**Figure 1:**
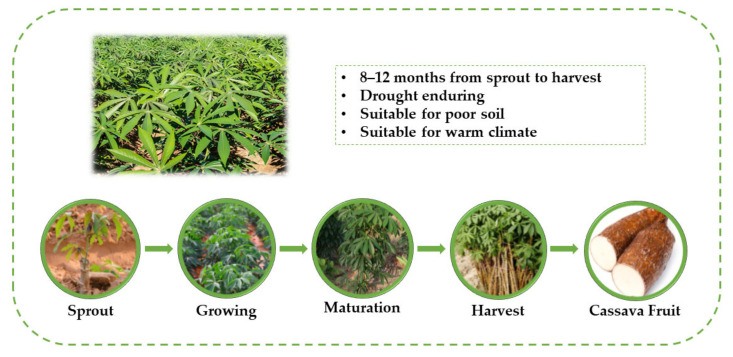
The field of cassava.

In China, the technology of biofuel ethanol production with corn and other foods as raw materials was mature and led to good economic benefits [20]. Corn ethanol production capacity accounted for 57% of the total [21]. However, with the increase in the population and fuel ethanol demand, competition emerged between raw materials, such as corn, and food security [22]. The Chinese government currently encourages the use of nonfood fuel ethanol to solve this conflict [23]. Compared with grain crops, cassava possesses low costs and a high capacity of ethanol production in the family of nongrain crops [24]. Cassava is one of the three main potato crops, originating in tropical America (Figure 1). Cassava has a low climate requirement, it can be grown in areas with high rainfall or dry land [25]. Tropical regions such as Nigeria, Thailand, Brazil, and southern China are major cassava producers. In 2002, the President of Nigeria proposed to develop cassava cultivation industry and promote cassava export [26]. Nigeria is the world’s largest producer of cassava. Brazil is the world’s third largest cassava producer, with a total output of 23.71 million tons and covering an area of 1.55 million hectares in 2016 [27]. In order to meet the targets of the Renewable Energy Development Plan, the Thailand Government has implemented a number of policies to support increased cassava production [28]. In China, cassava was first cultivated in the 19th century and mainly distributed in southern provinces such as Guangxi, Guangdong, and Yunnan [29]. Guangxi Province is the main cassava production area in China, with its cassava output accounting for more than 60% of the nation’s [30]; it is suitable for exploring the response mechanism of water quality to agriculture land-use changes.

In this study, a typical fuel ethanol raw material planting area was selected: a cassava planting area in the Guiping section of the Yujiang River Basin in Guangxi Province. The research content was mainly divided into the following three tasks:

(1) Land-use transformation patterns were revealed through the interpretation of remote sensing images for two terms.
(2) A nonpoint source pollution model was established after calibration and verification by a MIKE-SHE distributed hydrological model (Danish Hydraulic Institute, DHI, Copenhagen, Denmark). The influence of land-use changes on nonpoint source pollution in the watershed was obtained by multiple linear regression.
(3) A scenario hypothesis of ethanol crop cultivation was set up based on the results of step two, in order to guide the spatial layout of land-use planning in the fuel ethanol planting area.

This study divided the cultivated land into second-level classification and will provide scientific support and more elaborated management suggestions to local agricultural land-use planning. Furthermore, it could also set an example for other cassava planting areas worldwide for realizing the goal of carbon neutralization.

## 2. Materials and Methods

### 2.1. Overview of the Study Area

**Figure 2:**
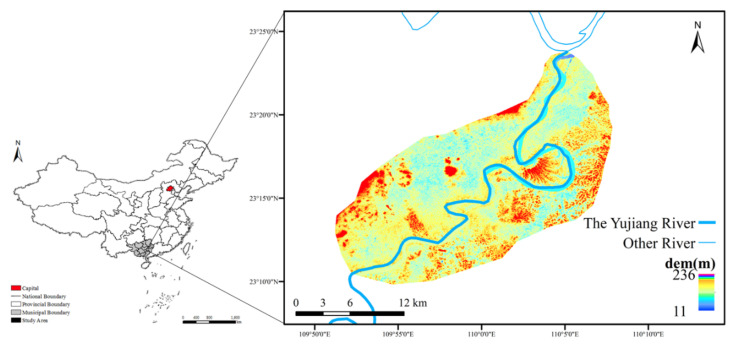
Overview of the cassava planting area of the Yujiang River Basin.

Guangxi Province is the largest cassava planting province in China. The Yujiang River is the largest tributary of the Xijiang River System in the Pearl River Basin. The Guiping section of the Yujiang River Basin has a total drainage area of 406.9 km^2^. The selected area is a typical raw material planting area for cassava fuel ethanol and contains a cassava plantation which area is 10 ha. Cassava planted in the basin is near the river, and it would help to reflect the influence of cassava planting structure change on nonpoint source pollution. The research takes place in the area where the Yujiang River flows from the southwest to the northeast, with a total length of about 45 km (Figure 2). The wet season in Guangxi is from May to October, and the mean flow season is from November to April of the next year.

**Figure 3:**
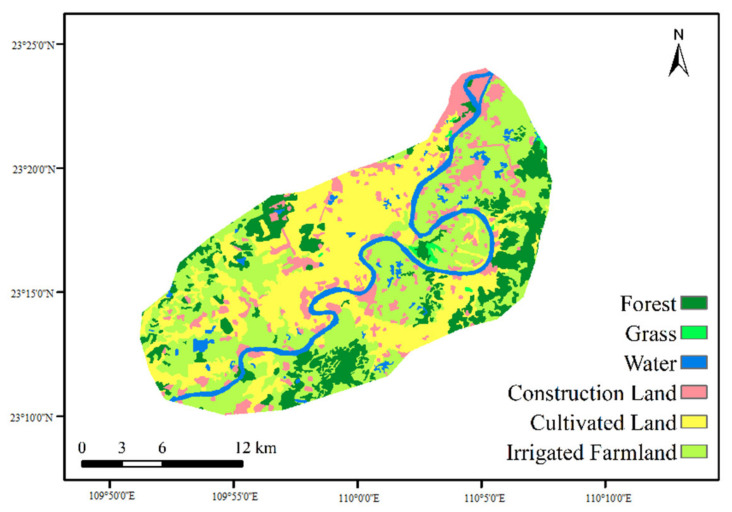
The distribution of land-use types in the cassava planting area.

The distribution of land-use types in the cassava planting area was shown in Figure 3, in which the occupied areas of cultivated land and irrigated farmland were 41.65% and 40.01%, respectively. The construction land accounted for 7.79%, and the river area was about 4.89%. In addition to cassava, there were maize and rice in this area. The soil types in the cassava planting area included rice soil, purple lime soil, yellow lateritic red soil, etc., of which acidic purple soil and yellow lateritic red soil occupied most of the area.

### 2.2. Interpretation of Land Use

Landsat-TM remote sensing image data with cloud volume ≤5% were downloaded in 2015 and 2020 from the official NASA website, and were preprocessed by radiation calibration, atmospheric correction, band synthesis, and image clipping. The results classified the land use into cultivated land, forest, grassland, water area, construction land, and unused land according to the classification system of the CAS (Chinese Academy of Sciences). The cultivated land in the Yujiang River Basin was further divided into cassava, corn, rice, and other cultivated land on the basis of different crop phenological information. The visualized spatial distribution maps of land-use types were displayed by raster data.

To reveal the internal transfer analysis of land use, a transfer matrix model was used as follows:

(1) $$A_{\mathrm{ij}}=\left[\begin{array}{c} \begin{array}{cc} A_{11} & A_{12}\\ A_{21} & A_{22} \end{array}\;\begin{array}{cc} \cdots & A_{1n}\\ \cdots & A_{2n} \end{array}\\ \begin{array}{cc} \vdots & \vdots \\ A_{n1} & A_{n2} \end{array}\;\begin{array}{cc} \ddots & \vdots \\ \cdots & A_{nn} \end{array} \end{array}\right]$$

where *A_ij_* was a matrix with *n* columns and *n* lines; *i* was the land-use type of the previous period, while *j* was that of the later period; *A_ij_* was the area transformed from type *i* to *j*; and *n* was the number of all of the land-use types.

### 2.3. Hydrological Model Construction

Compared with point source pollution, nonpoint source pollution had the characteristics of a complex mechanism and a wide spatial–temporal range. The estimation was also much more difficult than that of point source pollution. Therefore, a nonpoint source pollution model often assists in estimating the pollution load [31]. At present, the commonly used hydrological models include the SWAT model (United States Department of Agriculture, USDA, Washington, DC, USA) [32], AnnAGNPS model (USDA and Natural Resources Defense Council, New York, NY, USA) [33], MIKE model (DHI, Copenhagen, Denmark) [34], and so on. Among them, the MIKE-SHE model can simulate the hydrological response process of land-use change, and has more advantages in small- or medium-sized watersheds [35]. In this study, the MIKE-SHE model was selected as the simulation tool for the water quality analysis. The water quality simulation was based on the hydrodynamic model; the MIKE 11 module was selected to simulate the hydrodynamic situation. The MIKE-SHE model and MIKE 11 module were coupled based on the MIKE ZERO platform to simulate the water flow movement process in the river basin.

The input data required for the MIKE-SHE model simulation were listed in Table 1. TN and TP were measured by automatic water quality monitoring sensors and the data were obtained from Hydrology Centre of Guangxi Zhuangzu Autonomous Region.

## 3. Results and Discussion

### 3.1. Land-Use Interpretation Results

**Figure 4:**
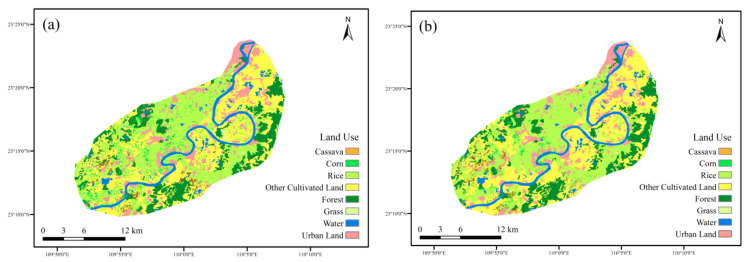
Land use of the Yujiang River Basin (**a**)—2015; (**b**)—2020.

The land-use types of the Yujiang River Basin included cassava, corn, rice, other cultivated land, forest, water area, and construction land. The interpretation results of the two terms are displayed in Figure 4.

Table 2 demonstrates the land-use area and proportion in the Yujiang River Basin from 2015 to 2020. The main land-use type was other cultivated land, with an average proportion of 34.5% of the two terms. Rice followed, with an average proportion of 29.8%. Cassava accounted for 1.4% and 1.5% of the total area, respectively. The area of cassava increased slightly from 2015 to 2020.

According to the land-use transfer matrix from 2015 to 2020 (Table 3), a transfer area with less than 0.05 km^2^ is denoted by 0.0 km^2^; the transfer area between land-use types was not obvious. The largest transfer crop was corn, with a total area of 14.7 km^2^, of which 46% was transferred to other cultivated land. The largest transferred area was other cultivated land, with a total area of 8.7 km^2^, of which 77% came from corn. From 2015 to 2020, cassava received 4 km^2^ of new area, of which 45% came from other cultivated land. Cassava transferred out a total of 3.8 km^2^, of which 53% was transferred to other cultivated land. This indicated that the conversion between cassava and other cultivated land was relatively easier than that of other types. Therefore, priority to the conversion between other cultivated land and cassava could be considered in future scenarios.

To sum up, the transfer matrix results could provide a theoretical basis for subsequent scenario assumptions. Other cultivated land could be the first choice to become cassava in the Yujiang River Basin.

### 3.2. Response Mechanism of Water Hydrology and Quality to Land-Use Changes

#### 3.2.1. Water Hydrology and Quality Simulation

**Figure 5:**
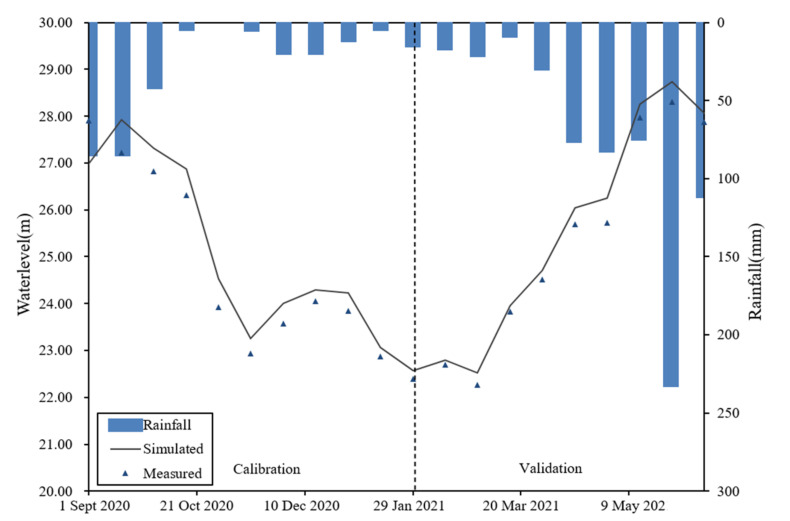
Measured and simulated water level values in the Yujiang River Basin.

Calibration period data of the Guiping Hydrological Station was selected from 1 September 2020 to 31 January 2021, and the verification period was set from 1 February 2021 to 13 June 2021 in the Yujiang River Basin. Through parameter adjustment, the final simulated and measured water level values were determined as shown in Figure 5.

The Nash coefficient of the Yujiang hydrodynamic model was 0.93; R^2^ was 0.95 in the calibration period. Those of the validation period were both 0.98, Nash coefficients >0.5 and R^2^ > 0.6, which indicated that the hydrodynamic model of the Yujiang River was credible and could reflect the real hydrodynamic situation.

As for the water quality, the simulation was based on the hydrodynamic results. The reliability was evaluated by the PBIAS (Position Bias) evaluation index. The smaller the PBIAS was, the smaller the deviation between the measured value and the simulated value. The water quality model can reflect the actual situation if the PBIAS is less than 25% [36].

**Figure 6:**
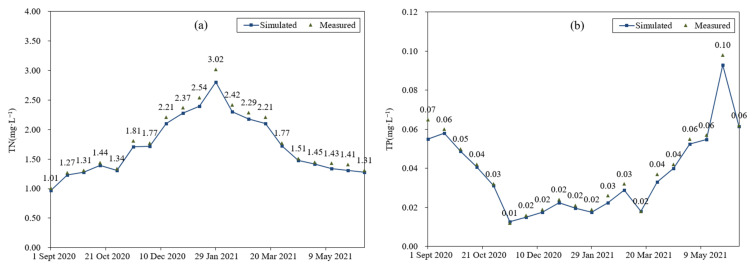
Measured and simulated TN (**a**) and TP (**b**) in the Yujiang River.

Figure 6 shows the comparison between the simulated and measured values of TN and TP at Baisha Station of the Yujiang River. The PBIAS indices of TN and TP were calculated to be 4.41% and 5.75%, respectively, indicating that the simulation effect was good and could accurately reflect the actual water quality change.

In the simulation period, the TN content exceeded the IV class of the surface water quality standard from 15 November 2020 to 13 April 2021. Since the dry season was from mid-November to March of the next year, the growth rate of aquatic plants was slow, and the water flowed steadily at the same time. The attenuation rate of pollutants reduced, resulting in an increase in nitrogen content while nonpoint source pollutants were scoured into the water body. After March, the water flow increased, accompanied with plant growth acceleration and an increase in rainfall. The pollutants’ diffusion and attenuation sped up, causing a decrease in the nitrogen content. In terms of the TP content, it performed better than the class II water quality of the surface water. The TP content began to decline in the middle of September, and to rise from November to June of the next year. The content of phosphorus in the water body was relatively small, and it mainly came from the surface runoff generated by the rainfall. Phosphorus was mainly adsorbed on soil particles. After rainfall events, the topsoil was eroded and entered the river, leading to an increase in the phosphorus content.

#### 3.2.2. Impact of Land-Use Changes on TN and TP Load

Based on the MIKE-SHE model simulation results in the Yujiang River Basin in 2015 and 2020, the Pearson correlation analysis between the land-use changes (with the samples *n* = 9) and TN as well as TP load was obtained (Table 4).

There was a very significant positive correlation between cultivated land change and TN and TP load (*p* = 0.978 **, 0.939 **); the same principle appeared in the construction land type. There was a very significant negative correlation between forest land change and pollutants (*p* = −0.945 **, −0.889 **), as was also the case with grassland (*p* = −0.881 **, −0.798 **). There was no remarkable correlation regarding the water area.

In conclusion, an increase in cultivated land and construction land would lead to a rise in TN and TP load, while an expansion in forest land and grassland area would reduce TN and TP load in the watershed.

Further efforts were made to the extent of crop structures. The Pearson correlation analysis is illustrated in Table 5 (with the samples *n* = 9). There was a very striking positive correlation between the changes in other cultivated land as well as corn area and TN as well as TP load (*p* = 0.851 **, 0.936 **) (*p* = 0.795 **, 0.826 **). The exerted impact of other cultivated land area was greater than that of corn land.

Multiple linear regression analysis was conducted to reveal the quantitative relations between crop structures and pollutant load. The main indexes are shown in Table 6.

The results stated clearly that corn and other cultivated land would have a striking positive impact on TN and TP, while rice and cassava would not have a momentous impact. The results were similar to the conclusions of some scholars. According to Guigang 2020 statistical yearbook, other cultivated land in the Yujiang River Basin was mainly divided into sugarcane and vegetables. In order to improve the yield, a large amount of nitrogen and phosphorus fertilizer needs to be applied. Sun’s research showed that growing corn requires a large amount of fertilization, which increased the risk of nonpoint source pollution [37]. The effects of other cultivated land changes on TN were greater than TP in this study. The result was the same as the result of Huang’s research. Huang found that the loss of P fertilizer was lower than that of N fertilizer under the same cropping pattern in southern China [38]. It was due to the hilly terrain in southern China exacerbated nitrogen loss from runoff. While the amount of fertilizer for cassava and rice was small, it had no remarkable effect on the load of TN and TP [39]. The result was the same as that of Jiang. Jiang found that the nonpoint source pollution in the basin would decline when growing tubers and rice instead of growing crops [40].

The low impact of cassava on nonpoint source pollution was due to the low fertilizer demand of cassava. It was suitable to plant in soil with low fertility. Cassava has a dense root system that could use nutrients efficiently. It made the cassava has less contribute to TN loads [41]. Cassava roots and mycorrhiza in soil could establish a symbiotic relationship [38]. This kind of symbiotic relationship enables cassava to absorb more TP than other crops. Patricia found that it could still ensure the cassava yield at lower fertilizer rate due to the storage of nitrogen and phosphorus by root system [42]. Therefore, applying less fertilizer to cassava could not only improves water quality, but also ensures cassava supply. The research results of the impact of crop structure changes on nonpoint source pollution would provide a theoretical basis for the subsequent land-use scenario hypothesis.

### 3.3. Land-Use Scenario Assumptions in the Yujiang River Basin

In order to reasonably speculate on the impact of land-use changes on the ethanol raw material planting area in the future, three scenarios were set up in the Yujiang River Basin. Land-use scenario assumption patterns mainly considered policy constraints, water quality constraints, and economic benefit constraints, among which water quality constraints referred to the relation coefficients presented in Section 3.2.2.

To achieve the goal of carbon neutrality as soon as possible, the Chinese government cancelled the preferential tax policy for ethanol production with grain crops as raw materials since 2016, and implemented a subsidy plan for nongrain fuel ethanol production to encourage the use of fuel ethanol production based on nongrain crops. In the future, nongrain biofuel ethanol would develop vigorously [43]. China’s national energy administration proposed focusing on promoting the nongrain biofuel ethanol industry in 2021. Therefore, it is necessary to expand the planting area of cassava in Guangxi Province or in other regions, which would help to accelerate the promotion of the cassava fuel ethanol industry under the energy policy framework. Since the increase in other cultivated land had a negative impact on the water quality, while the increase in cassava had no significant impact, the scenario hypothesis of increasing cassava could be set under the water quality constraints. As to the economic aspect, a cost–benefit calculation was carried out to ensure farmers’ income and economic benefits.

Three scenarios were set up, which are presented below:

(1) Scenario One: According to the results of a previous loss risk assessment of the Yujiang River Basin, the intersection of high-risk loss areas of nitrogen as well as phosphorus and other cultivated land was set to be converted into a cassava area.
(2) Scenario Two: The intersection of medium- and high-risk loss areas of nitrogen as well as phosphorus and other cultivated land was set to be converted into a cassava area.
(3) Scenario Three: The cassava area continuously increased until the profit of other cultivated land (sugarcane and vegetables) was replaced by the cassava benefit difference. According to the economic data obtained in 2020, the upper limit for the cassava area was 126.0 km^2^. The area of corn and rice would remain unchanged to ensure food security.

**Figure 7:**
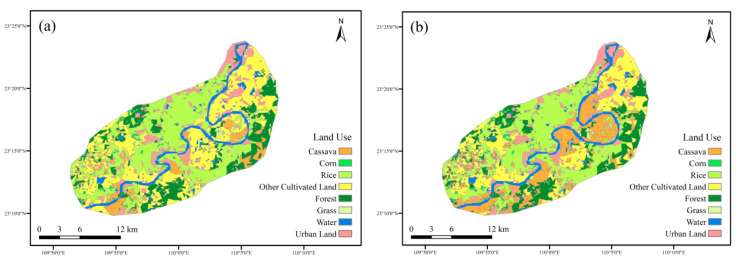

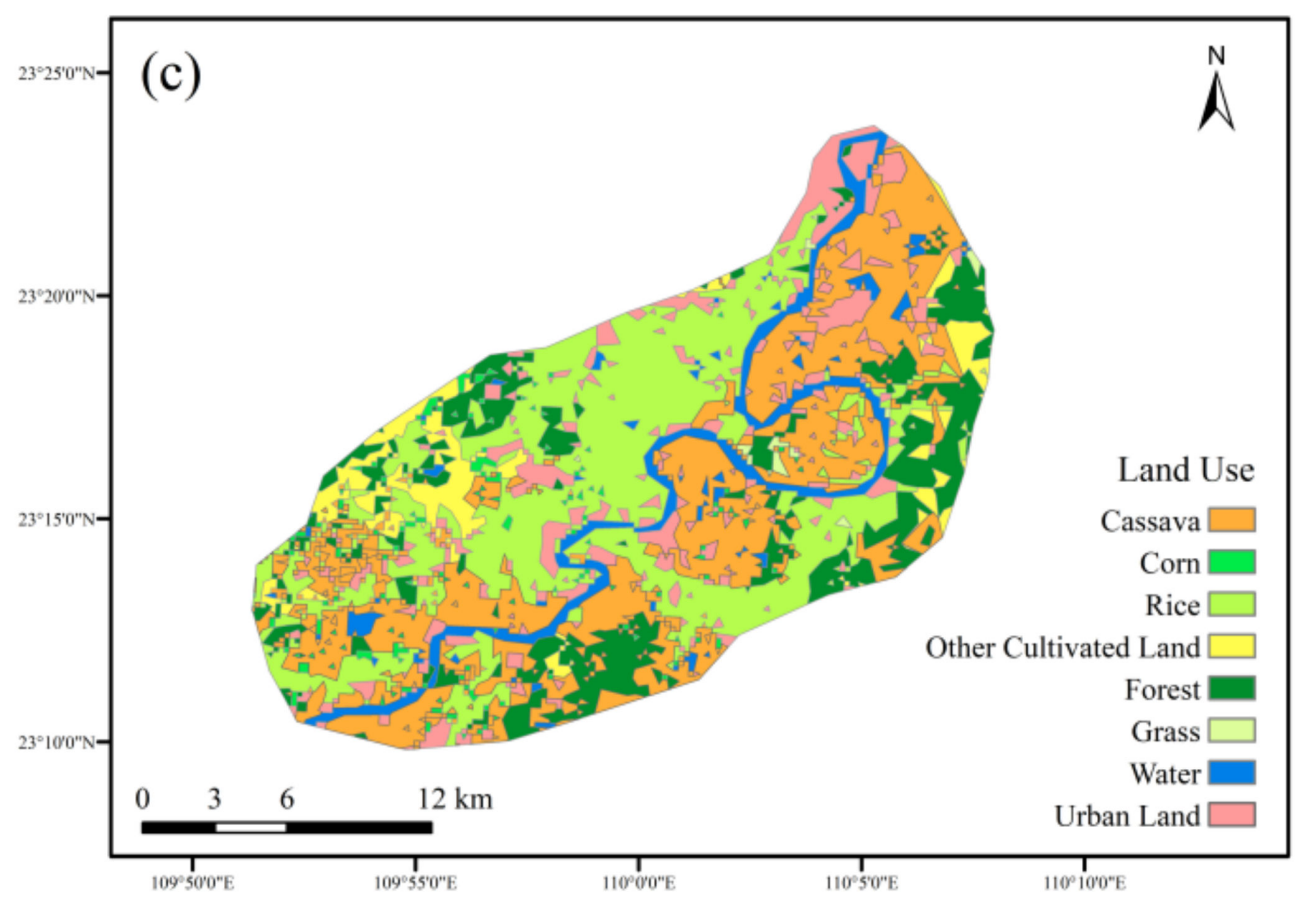
The scenarios of land use (**a**)—scenario one; (**b**)—scenario two; and (**c**)—scenario three.

These scenarios of land use are displayed in Figure 7.

Table 7 shows the land-use changes under the three scenarios.

**Figure 8:**
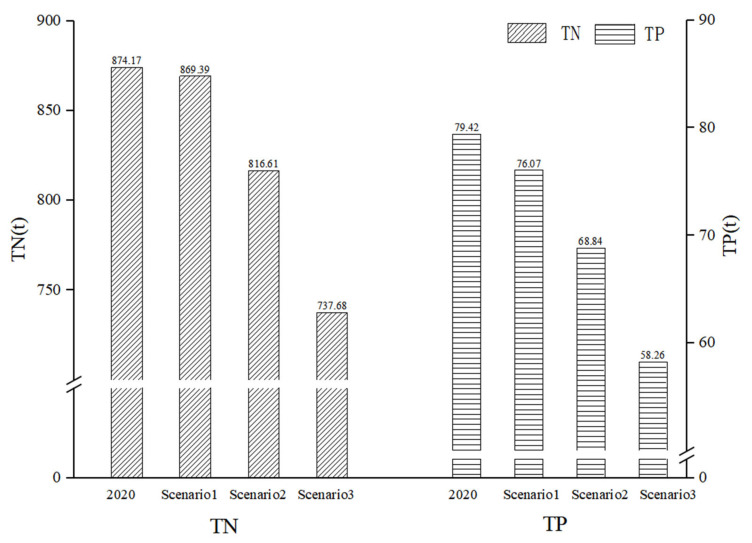
TN and TP loads at the basin outlet of the different scenarios.

Taking the land-use distribution under the three scenarios into the validated hydrological model, the TN loads at the basin outlet were 869.39 tons, 816.61 tons, and 737.68 tons, respectively. Meanwhile, the TP loads were 76.07 tons, 68.84 tons, and 58.26 tons, respectively (Figure 8). The results revealed that the cassava area replacing other cultivated land could assist in reducing the nonpoint source pollution load.

In addition to fertilization, planting patterns also affected nonpoint source pollution loads. Single cropping patterns could lead to soil fertility decline and destroy the microbial community. It would reduce crop yields and increase the risk of nonpoint source pollution while crop rotation system could effectively reduce nitrogen and phosphorus loss caused by fertilization [44]. Jiang found that planting potatoes in spring and peanuts in autumn could not only ensure the grain yield and economic benefits of farmers, but also minimize the nitrogen load [39]. Another study found that cassava grew slowly in the initial stage, but the soil coverage was low, which was suitable for intercropping with beans with a fast growth cycle. It would increase yield for cassava and ensure farmers’ economic income [45].

The development of cassava fuel ethanol could be a conducive way to improve the quality of the water environment and realize this goal from a low-carbon perspective. In addition, the increase in fuel ethanol demand might promote an increase in cassava production and demand, which could lead to a rise in its price. The leveling up of the cassava price could uplift farmers’ income and reduce the profit gap with other economic crops. On the other hand, this change may affect the interests of cassava ethanol production companies. In order to eliminate the negative impact, appropriate agricultural policies are required to balance the demand and supply of cassava ethanol. Therefore, the state should provide more aid in terms of agricultural and energy incentives for cassava planting and cassava ethanol production.

## 4. Conclusions

This study selected a typical fuel ethanol planting area in South China to explore the land-use changes that have occurred in recent years. Combined with the typical crop structure changes in the local area, the response mechanism of water quality was revealed by using the distributed hydrological model. Based on the quantitative relations coefficients, scenarios under the carbon neutralization policy were carried out to forecast the nonpoint source pollutants of the river.

(1) The main land-use type in the Yujiang River Basin was other cultivated land, while the area of cassava is increasing. From the land-use transition matrix, the conversion between cassava and other cultivated land was the easiest, which gave a case principle for the scenario’s assumption setting.
(2) The increase in cultivated land and construction land would lead to a rise in the load of TN and TP, while an expansion of forest land and grassland area would reduce TN and TP load in the watershed. As for the crop structures, corn would have a significant positive impact on TN and TP, while rice and cassava would not have a striking impact.
(3) The increase in the cassava area in the Yujiang River Basin was beneficial to reduce nonpoint source pollution. The maximum increase in the area of cassava should be 126 km^2^. If it continues to rise past that level, it could cause negative impacts on farmers’ income and economic benefits.

In summary, the cassava planting area had little influence on NPS pollution in the Yujiang River Basin, which was suitable for the vigorous expansion of fuel ethanol. The threshold for cassava extension was suggested after the scenario analysis. In order to reduce the impact of cassava nonpoint source pollution, managers could adopt rotation and intercropping. Intercropping cassava and crops with shorter growth cycles could effectively reduce the risk of nonpoint source pollution. Tropical countries such as Nigeria, Brazil, and Thailand could choose to promote cassava cultivation. Meanwhile, the introduction of cassava ethanol industry in the local area could improve the national economic efficiency and ensure the global clean energy supply. This research could provide scientific support for local agriculture land-use management to realize the carbon neutralization vision and also set a good example for the development of the cassava fuel ethanol industry in other cassava-planting countries, such as Thailand, Nigeria, and Indonesia.

## Figures and Tables

**Table 1:** The data required in the MIKE-SHE model.

Data Types	Name	Data Source
Geographical data	DEM elevation data	GS Cloud
Hydrological data	River network River section Discharge Water level TN and TP concentration	Hydrology Center of Guangxi Zhuangzu Autonomous Region (http://swzx.gxzf.gov.cn/) (accessed on 10 October 2021)
Water quality data	Fertilizer	2020 National Agricultural Product Cost-benefit Data Corpus (https://www.yearbookchina.com/navibooklist-n3020013195-1.html) (accessed on 1 September 2021)
Meteorological data	Precipitation Reference evapotranspiration	National Meteorological Science Data Center (http://data.cma.cn/) (accessed on 15 September 2021)
Vegetation	Leaf area index Root depth	The literature surveys FAO (https://www.fao.org/land–water/databases–and–software/crop–information/en/) (accessed on 15 October 2021)
Soil properties	Surface and sectional type	Harmonized World Soil Database

**Table 2:** Land-use area and proportion of the Yujiang River Basin (km^2^).

Type		Cassava	Corn	Rice	Other Cultivated Land	Forest	Grass	Water	Urban Land
2015	Area/km^2^	5.6	12.3	121.1	138.8	56.7	1.7	25.5	45.1
	Percentage	1.4%	3.0%	29.8%	34.1%	13.9%	0.4%	6.3%	11.1%
2020	Area/km^2^	6.1	6.7	121.5	142.1	58.1	1.7	26.7	43.9
	Percentage	1.5%	1.7%	29.9%	34.9%	14.3%	0.4%	6.6%	10.8%

**Table 3:** Land-use transfer matrix of the Yujiang River Basin (km^2^).

2015 2020	Cassava	Corn	Rice	Other Cultivated Land	Forest	Grass	Water	Urban Land
Cassava	2.3	0.7	0.7	2.0	0.2	0.0	0.1	0.1
Corn	0.5	1.5	5.2	6.7	1.4	0.0	0.3	0.6
Rice	1.1	2.0	113.0	0.0	0.0	0.0	0.0	0.0
Other Cultivated Land	1.8	2.6	0.0	130.1	0.0	0.0	0.0	0.0
Forest	0.3	0.9	0.0	0.0	56.5	0.0	0.0	0.0
Grass	0.0	0.0	0.0	0.0	0.0	1.9	0.0	0.0
Water	0.1	0.1	0.0	0.0	0.0	0.0	27.1	0.0
Urban Land	0.2	0.2	0.0	0.0	0.0	0.0	0.0	45.3

**Table 4:** Pearson correlation analysis between the land-use changes and TN/TP load.

	Cultivated Land	Forest	Grass	River	Urban Land
TN	0.978 **	−0.945 **	−0.881 **	0.185	0.901 **
TP	0.939 **	−0.889 **	−0.798 **	0.078	0.912 **

** *p* < 0.01: the correlation was significant at the level of 0.01 (bilateral).

**Table 5:** Pearson correlation analysis between crop changes and TN/TP load.

	Corn	Rice	Cassava	Other Cultivated Land
TN	0.795 *	0.504	–0.351	0.851 **
TP	0.826**	0.318	–0.353	0.936 **

** *p* < 0.01: the correlation was significant at the level of 0.01 (bilateral); * *p* < 0.05: the correlation was significant at the level of 0.05 (bilateral).

**Table 6:** Multiple linear regression analysis of TN and TP of different crops.

	TN		TP	
	B	*p*	B	*p*
Cassava	4.699	0.097	0.108	0.307
Corn	3.349 *	0.030 *	0.180	0.017 *
Rice	2.712	0.099	0.072	0.226
Other cultivated land	3.659 **	0.009 **	0.262	0.012 *
R^2^	0.967		0.985	
F	F = 29.606, *p* = 0.003		F = 63.791, *p* = 0.001	

** *p* < 0.01: the correlation was significant at the level of 0.01 (bilateral). * *p* < 0.05: the correlation was significant at the level of 0.05 (bilateral).

**Table 7:** Areas of the land-use scenarios in the Yujiang River Basin (km^2^).

Types	2020	Scenario One		Scenario Two		Scenario Three	
	Area	Area	Change	Area	Change	Area	Change
Cassava	6.1	25.0	+308%	66.0	+978%	126.0	+1958%
Corn	6.7	6.7	0%	6.7	0%	6.7	0%
Rice	121.5	121.5	0%	121.5	0%	121.5	0%
Other cultivated land	142.1	123.2	−13%	82.2	−42%	22.2	−84%
Forest	58.1	58.1	0%	58.1	0%	58.1	0%
Grass	1.7	1.7	0%	1.7	0%	1.7	0%
Water	26.7	26.7	0%	26.7	0%	26.7	0%
Urban land	43.9	43.9	0%	43.9	0%	43.9	0%

## Data Availability

The data presented in this study are available on request from the corresponding author.

## References

[1] International Energy Agency (2018). World Energy Outlook 2018.

[2] Sharma S., Kundu A., Basu S., Shetti N.P., Aminabhavi T.M. (2020). Sustainable environmental management and related biofuel technologies. J. Environ. Manag..

[3] European Commission Sustainable Transport|Mobility and Transport. https://ec.europa.eu/transport/themes/sustainable_en.

[4] Richard C.J., Josep M.M.S., Jordi G., Laureano J., Carlos P. (2022). Comparing biofuels through the lens of sustainability: A data envelopment analysis approach. Appl. Energy..

[5] Mahmood E., Susan V.D., James D.M., Jack S. (2020). Biofuels policies that have encouraged their production and use: An international perspective. Energy Policy.

[6] Teklit G.A., Mentore V., Adrián B.P., Shiv P., Eric D.V.H., Sami R.J. (2021). Emerging technologies for biofuel production: A critical review on recent progress, challenges and perspectives. J. Environ. Manag..

[7] Cameron H., Qi Y., Nicholas S., Bob W., Xie C., Dimitri Z. (2021). Towards carbon neutrality and China’s 14th Five–Year Plan: Clean energy transition, sustainable urban development, and investment priorities. Environ. Sci. Technol..

[8] Austin K.G., Jones J.P.H., Clark C.M. (2022). A review of domestic land use change attributable to U.S. biofuel policy. Renew. Sustain. Energy Rev..

[9] Fadhil K.J., Katherine G. (2019). Statistical assessment of nonpoint source pollution in agricultural watersheds in the Lower Grand River watershed, MO, USA. Environ. Sci. Pollut. Res..

[10] Cui G., Wang X., Li C., Li Y., Yan S., Yang Z. (2018). Water use efficiency and TN/TP concentrations as indicators for watershed land-use management: A case study in Miyun District, north China. Ecol. Indic..

[11] Yan S., Wang X., Cai Y., Li C., Yan R., Cui G., Yang Z. (2018). An Integrated Investigation of Spatiotemporal Habitat Quality Dynamics and Driving Forces in the Upper Basin of Miyun Reservoir, North China. Sustainability.

[12] Xu Y., Wang X., Bai J., Wang D., Wang W., Guan Y. (2020). Estimating the Spatial Distribution of Soil Total Nitrogen and Available Potassium in Coastal Wetland Soils in the Yellow River Delta by Incorporating Multi-Source Data. Ecol. Indic.

[13] Moriken C., Nor R.J., Ahmad F.B.A. (2019). Impact of land uses on water quality in Malaysia: A review. Ecol. Process..

[14] Ni X., Parajuli P.B., Ouyang Y., Dash P., Siegert C. (2021). Assessing land use change impact on stream discharge and stream water quality in an agricultural watershed. CATENA.

[15] Chen Y., Ale S., Rajan N., Srinivasan R. (2017). Modeling the effects of land use change from cotton (Gossypium hirsutum L.) to perennial bioenergy grasses on watershed hydrology and water quality under changing climate. Agric. Water Manag..

[16] Rebecca L.R., Nathaniel R.S., Gail T. (2009). Identifying potential environmental impacts of large–scale deployment of dedicated bioenergy crops in the UK. Renew. Sust. Energ. Rev..

[17] Liu Y., Cui G., Bai X., Yu Z., Dong L. (2021). Characteristics and source apportionment of nitrogen and phosphorus non-point source pollution in Wuming River Basin, Guangxi. J. China Environ. Sci..

[18] Roy R.G., Mahesh K.S., Manoj K.J. (2015). Simulating the impacts of bio–fuel crop production on nonpoint source pollution in the Upper Mississippi River Basin. Ecol. Eng..

[19] Ouyang W., Hao X., Wang L., Xu Y., Mats T., Gao X., Lin C. (2019). Watershed diffuse pollution dynamics and response to land development assessment with riverine sediments. Sci. Total Environ..

[20] Nurul S.M.A., Kuan S.K., Kit W.C., Pau L.S., Chen W.H., Hong P.N. (2020). Sustainability of the four generations of biofuels—A review. Int. J. Energy Res..

[21] Zhang Z., Li G., Zhang Y., Zhang J., Song C., Zhou Y. (2020). Recommendations for green development of motor biofuel industry in China: A review. Int. J. Agric. Biol. Eng..

[22] Boly M., Sanou A. (2022). Biofuels and food security: Evidence from Indonesia and Mexico. Energy Policy.

[23] Azhaham P.S., Arivalagan P., Thangavel M. (2020). A comprehensive assessment of biofuel policies in the BRICS nations: Implementation, blending target and gaps. Fuel.

[24] Chantima R., Seksan P., Ruethai O., Benjamaporn T. (2021). Evaluation of the environmental performance of bioethanol from cassava pulp using life cycle assessment. J. Clean. Prod..

[25] Enesi R., Hauser S., Pypers P., Kreye C., Tariku M., Six J. (2022). Understanding changes in cassava root dry matter yield by different planting dates, crop ages at harvest, fertilizer application and varieties. Eur. J. Agron..

[26] Donkor E., Onakuse S., Bogue J., Bogue J., Carmenado I.D. (2017). The impact of the presidential cassava initiative on cassava productivity in Nigeria: Implication for sustainable food supply and food security. Cogent. Food Agric..

[27] Visses F., Sentelhas P.C., Pereira A.B. (2018). Yield gap of cassava crop as a measure of food security—An example for the main Brazilian producing regions. Food Secur..

[28] Jakrawatana N., Pingmuangleka P., Gheewala S.H. (2016). Material flow management and cleaner production of cassava processing for future food, feed and fuel in Thailand. J. Clean. Prod..

[29] Luo C., Yang L., Ou Z., Luo Y. (2019). Current Situation and Prospects of Cassava Food Processing. J. Acta Agric. Jiangxi.

[30] Hao M., Jiang D., Wang J., Fu J., Huang Y. (2017). Could biofuel development stress China’s water resources. GCB Bioenergy.

[31] Li H.Z., Zhang M.X. (2019). A review on the calculation of non—Point source pollution loads. IOP Conf. Ser. Environ. Earth Sci..

[32] Hong H.N., Friedrich R., Wayne M., Jacqueline F., He Y., Matthew S.G. (2019). Comparison of the alternative models SOURCE and SWAT for predicting catchment streamflow, sediment and nutrient loads under the effect of land use changes. Sci. Total Environ..

[33] Yasarer L.M.W., Lohani S., Bingner R.L., Locke M.A., Baffaut C., Thompson A.L. (2019). Assessment of the Soil Vulnerability Index and comparison with AnnAGNPS in two Lower Mississippi River Basin watersheds. J. Soil Water Conserv..

[34] Liu S., Liu H., Wang L. (2018). Research on the development and application of Mike she model. J. China Hydrol..

[35] Thanh T.N., Ingrid K., Patrick W. (2018). Conceptual river water quality model with flexible model structure. Environ. Model. Softw..

[36] Sun C., Chen L., Zhai L., Liu H., Wang K., Jiao C., Shen Z. (2020). National assessment of nitrogen fertilizers fate and related environmental impacts of multiple pathways in China. J. Clean. Prod..

[37] Huang J., Xu C., Ridoutt B.G., Wang X., Ren P. (2017). Nitrogen and phosphorus losses and eutrophication potential associated with fertilizer application to cropland in China. J. Clean. Prod..

[38] Pypers P., Bimponda W., Lodi L., Jean P., Lele B., Mulumba R., Kachaka C., Boeckx P., Merckx R., Vanlauwe B. (2012). Combining Mineral Fertilizer and Green Manure for Increased, Profitable Cassava Production. Agron. J..

[39] Jiang J., Li J., Wang Z., Wu X., Lai C., Chen X. (2022). Effects of different cropping systems on ammonia nitrogen load in a typical agricultural watershed of South China. J. Contam. Hydrol..

[40] Adiele J.G., Schut A.G.T., van den Beuken R.P.M., Ezui K.S., Pypers P., Ano A.O., Egesi C.N., Giller K.E. (2020). Towards closing cassava yield gap in West Africa: Agronomic efficiency and storage root yield responses to NPK fertilizers. Field Crops Res..

[41] Howeler R.H. (2002). Cassava: Biology, Production and Utilization.

[42] Li Z. (2020). Evaluation of Water Pollution Control Measures in Yanghe Basin Based on MIKE SHE Model. Master’s Thesis.

[43] Hao H., Liu Z., Zhao F., Ren J., Chang S., Rong K., Du J. (2018). Biofuel for vehicle use in China: Current status, future potential and policy implications. Renew. Sust. Energ. Rev..

[44] Ding H., Ali A., Cheng Z. (2018). Dynamics of a Soil Fungal Community in a Three-Year Green Garlic/Cucumber Crop Rotation System in Northwest China. Sustainability.

[45] Silva D.V., Ferreira E.A., Oliveira M.C., Pereira G.A.M., Braga R.R., Santos J.D., Aspiazu I., Souza M.F. (2016). Productívíty of cassava and other crops in an intercropping system. Cienc. Investig. Agrar..

